# Functional siRNA Delivery via Jet Nebulization: Proof-of-Concept IL-1ß Silencing in Macrophage-like THP-1 Cells

**DOI:** 10.3390/ijms27062915

**Published:** 2026-03-23

**Authors:** Duy Bao Tran Nguyen, Ahmed S. M. Ali, Dongwei Wu, Johanna Berg, Daniel C. Lauster, Jens Kurreck, Beatrice Tolksdorf

**Affiliations:** 1 Department of Applied Biochemistry, Institute of Biotechnology, Technische Universität Berlin, 10623 Berlin, Germany; 2 Institute of Pharmacy, Biopharmaceuticals, Freie Universität Berlin, 12169 Berlin, Germany

**Keywords:** siRNA, nebulization, IL-1β knockdown, aerosolized delivery, inhaled RNAi, jet nebulizer

## Abstract

The efficient delivery of small interfering RNAs (siRNAs) to immune and respiratory cells represents a key methodological challenge in developing inhaled RNA interference (RNAi) approaches. A central question is whether siRNA functionality is preserved following aerosolization, as the mechanical stress of nebulization may compromise siRNA integrity and silencing activity. Here, we report a proof-of-concept study using THP-1-derived macrophage-like cells as a tractable in vitro model to characterize jet nebulization for siRNA delivery. Three siRNA candidates targeting interleukin-1 beta (IL-1β) were computationally designed and validated for potent silencing activity and low cytotoxicity. Using a commercially available, off-the-shelf jet nebulizer combined with Lipofectamine RNAiMAX, we demonstrate that siRNA-lipoplexes retain their gene-silencing activity after aerosolization, achieving robust IL-1β knockdown. The delivery efficiency was influenced by siRNA-lipoplex complexation, highlighting the importance of formulation parameters. These findings establish a practical and accessible in vitro platform for evaluating nebulized siRNA functionality, providing a foundation for future studies in more complex and physiologically relevant airway models.

## 1. Introduction

Chronic inflammation is a major driver of numerous pathological conditions, including autoimmune diseases, metabolic disorders, chronic lung diseases, and cancer [1]. Beyond infection defense, persistent inflammation contributes to disease progression through immune dysregulation, tissue remodeling, and altered cellular signaling [2,3,4,5]. Among the central mediators, the pro-inflammatory cytokine Interleukin-1 beta (IL-1β) plays a pivotal role in initiating and amplifying immune responses [6,7].

IL-1β is mainly produced by activated macrophages in response to pathogenic stimuli and cellular stress, driving fever induction, immune cell recruitment, and tissue remodeling [7,8]. By binding to the IL-1 receptor on target cells, IL-1β activates nuclear factor-κB and mitogen-activated protein kinase pathways, leading to the transcription of inflammatory mediators, including other cytokines, chemokines, adhesion molecules, and proteolytic enzymes that sustain inflammation [8,9,10]. While acute inflammation is protective, facilitating immune cell recruitment and tissue repair, dysregulated IL-1β signaling contributes to chronic inflammatory diseases, such as rheumatoid arthritis, inflammatory bowel disease, and chronic obstructive pulmonary disease, and cancer [11,12,13,14,15].

While monoclonal antibodies and small-molecule inhibitors targeting IL-1β have been developed and approved for clinical use [16,17,18], their application is often limited by high costs, systemic immunosuppression, and potential immunogenicity [19]. As an alternative, RNA interference (RNAi) offers a promising approach to silence IL-1β mRNA [20,21], leading to decreased IL-1β protein synthesis. RNAi is a naturally occurring post-transcriptional gene regulation mechanism that enables the precise and sequence-specific degradation of target mRNA [22]. Small interfering RNAs (siRNAs) mediate RNAi by binding to complementary mRNA sequences, recruiting the RNA-induced silencing complex, and guiding the cleavage and degradation of the target transcript [23]. Treatment with siRNAs thereby prevents synthesis of the encoded protein. This mechanism provides high specificity, efficiency, and durability relative to traditional inhibitors, making RNAi a powerful tool for gene silencing [24,25].

A central methodological challenge in RNAi-based research is the efficient delivery of siRNA while maintaining their functionality, stability, and cellular uptake. Lipid-based reagents (e.g., Lipofectamine) are commonly used for in vitro delivery [26,27]. However, their systemic administration is limited by toxicity and biodistribution [28,29,30]. Aerosolized drug delivery via nebulization offers a non-invasive approach, allowing for the direct administration of siRNA formulations to respiratory tissues and immune cells in the lung [31,32,33]. This localized delivery strategy has potential for treating respiratory inflammatory conditions and warrants further methodological investigation. Recent preclinical studies have demonstrated the therapeutic efficacy of nebulized siRNA in animal models. Meng et al. achieved significant bacterial clearance and gene silencing in a murine pneumonia model using a lipid-based siRNA formulation [34], while Dong et al. reported attenuated fibrosis following inhaled nanoparticle-mediated delivery of siRNA targeting interleukin-11 in a murine bleomycin-induced lung injury model [35]. These advances highlight the broader potential of nebulization as an siRNA delivery route. At the same time, simple and accessible in vitro platforms using standard equipment offer a practical complement for early-stage characterization of siRNA functionality under nebulization conditions, particularly in the context of ongoing efforts to reduce reliance on animal models [36].

In this methodological proof-of-concept study, we established a defined and tractable in vitro system to characterize siRNA functionality following jet nebulization. THP-1-derived macrophage-like cells treated with lipopolysaccharide (LPS) were used to model acute inflammatory activation, a well-established and widely used model for investigating inflammatory processes and immune responses [37,38]. LPS stimulation was applied to induce IL-1β expression, providing a quantifiable functional readout of siRNA activity post-nebulization. We assessed IL-1β knockdown by nebulizing siRNA-cationic lipid complexes (siRNA-lipoplexes) using a commercially available, off-the-shelf jet nebulizer combined with a custom-made setup for direct aerosol deposition and compared their performance to conventional forward transfections. This in vitro feasibility study provides a practical and accessible platform for the systematic characterization of nebulization parameters and confirms siRNA stability and functionality following aerosolization. Future studies employing primary macrophages, mixed airway cell cultures, and three-dimensional airway models will further extend this approach toward more physiologically relevant systems.

## 2. Results

### 2.1. Design of siRNAs Targeting IL-1β and Evaluation of Silencing Efficiency

Potent siRNA sequences targeting IL-1β were identified through computational screening based on thermodynamic stability, GC content, and predicted target site accessibility [39,40]. Secondary structure analysis of IL-1β mRNA using mFold (Figure S1) supported the selection of three siRNAs (siIL-1β.1, siIL-1β.2, siIL-1β.3) targeting structurally accessible regions [41]. Off-target effects were minimized by excluding seed matches in the human transcriptome using BLASTn 2.14.1. A scrambled siRNA (siCon) lacking homology to any human sequence was used as a negative control [42].

**Figure 1:**
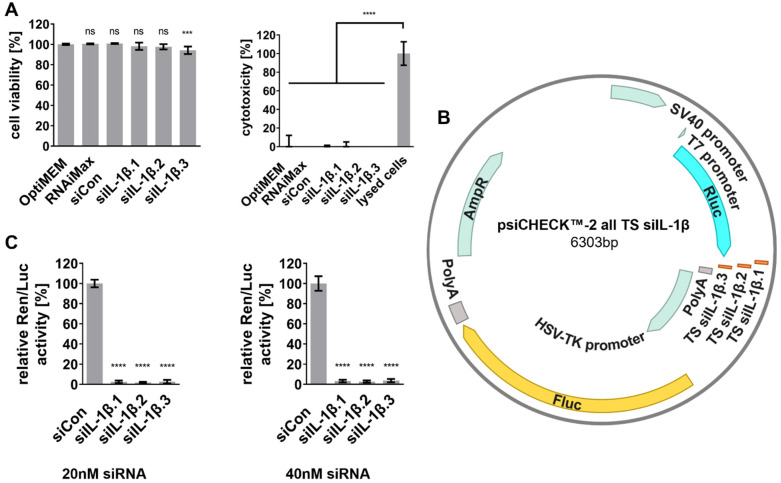
Evaluation of siRNA cytotoxicity and silencing efficiency. (**A**) XTT assay results indicating cell viability (**left**) and LDH assay results measuring cytotoxicity (**right**) in THP-1 Mφ treated with 40 nM of siRNA candidates (siIL-1β.1, siIL-1β.2, siIL-1β.3) or controls (transfection medium Opti-MEM, transfection reagent RNAiMAX, and siCon). (**B**) Schematic of the psiCHECK™-2 dual-luciferase reporter plasmid. *Renilla* luciferase (Rluc) serves as the primary reporter, with siRNA target sequences (TS) cloned downstream of the reporter gene. Firefly luciferase (Fluc) is co-expressed for normalization. (**C**) Relative Ren/Luc activity in HeLa cells co-transfected with siRNAs at 20 nM (**left**) or 40 nM (**right**). Relative luciferase activity was normalized to siCon set as 100%. Data are represented as mean ± SD from three independent experiments (n = 3), with statistical significance denoted as *** *p* ≤ 0.001, **** *p* ≤ 0.0001, and ns (non-significant, *p* > 0.05). Statistical analysis was performed using one-way ANOVA followed by Dunnett’s multiple comparison test.

The potential cytotoxicity of the designed siRNAs was evaluated using 2,3-bis-(2-methoxy-4-nitro-5-sulfophenyl)-2H-tetrazolium-5-carboxanilide (XTT) and lactate dehydrogenase (LDH) release assays. For this, monocytic THP-1 cells were differentiated with phorbol 12-myristate 13-acetate (PMA) into adherent macrophage-like cells (THP-1 Mφ). Subsequently, the THP-1 Mφ were transfected with the designed siRNAs. All siRNA-treated cultures, including siIL-1β.1, siIL-1β.2, and siIL-1β.3, exhibited cell viability >95% (Figure 1A). Only siIL-1β.3 displayed a slight but statistically significant reduction in viability, while LDH assays confirmed that there were no cytotoxic effects in any culture compared to controls.

The silencing activity of the siRNAs was assessed in initial experiments using dual-luciferase assays (DLAs) in HeLa cells, which are established for RNAi studies due to their high transfection efficiency [43]. The reporter plasmid (psiCHECK™-2 all TS siIL-1β, Figure 1B), containing the target sequences for each siRNA fused to the *Renilla* luciferase gene (Rluc), was co-transfected with the respective siRNAs into HeLa cells. The firefly luciferase gene (Fluc) served as an internal control for transfection normalization.

Significant silencing activity was observed for all three siRNA candidates (siIL-1β.1, siIL-1β.2, siIL-1β.3) at both 20 nM and 40 nM concentrations. At 40 nM, relative Ren/Luc activity was reduced by at least 97% for all siRNAs compared to the negative control (siCon), while similar silencing activity was maintained at 20 nM (Figure 1C). These results highlight the robust silencing potential of the designed siRNAs, even at the lower concentration of 20 nM, making them suitable for further knockdown experiments.

### 2.2. Knockdown of Endogenous IL-1β Protein Levels

Endogenous IL-1β protein reduction served as the primary functional readout of siRNA-mediated gene silencing. THP-1 Mφ were forward-transfected with 20 nM siRNAs and stimulated with 100 ng/mL LPS to induce IL-1β production, simulating an inflammatory response.

**Figure 2:**
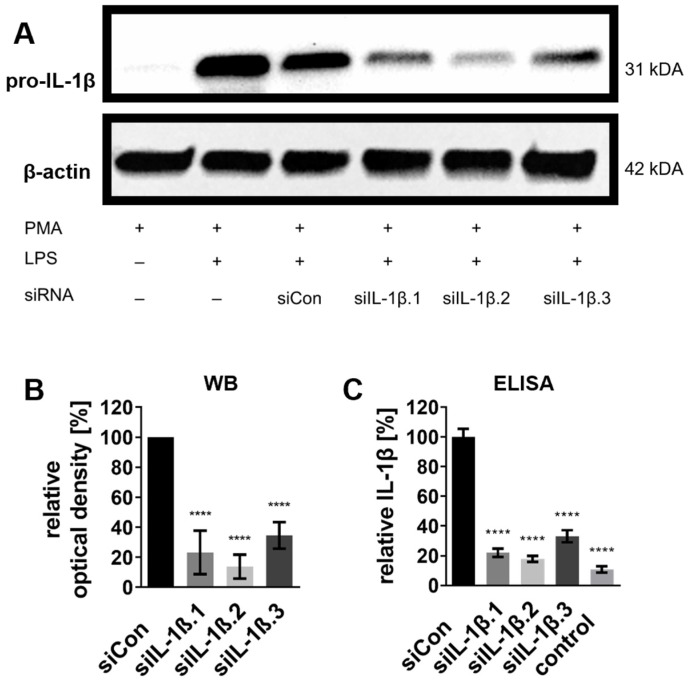
IL-1β protein knockdown in differentiated THP-1 cells post-siRNA transfection. Cells were treated with 100 ng/mL PMA for 72 h to induce differentiation into THP-1 Mφ. Following differentiation, cells were stabilized in PMA-free medium for 24 h before transfection with 20 nM siRNA. At 43 h post-transfection, cells were stimulated with 100 ng/mL LPS for 5 h to induce an immune response. Non-stimulated, non-transfected THP-1 Mφ served as the control condition. (**A**) Representative Western blot showing pro-IL-1β protein expression under various treatment conditions, including the addition (+) or absence (−) of PMA, LPS, and siRNA transfection. β-actin served as a loading control to confirm equal protein loading (20 μg per lane). (**B**) Densitometric quantification of pro-IL-1β protein levels from Western blots, normalized to β-actin and expressed relative to siCon set as 100%. (**C**) Relative IL-1β protein levels quantified by ELISA, normalized to siCon set as 100%. Data are presented as mean ± SD from three independent experiments (n = 3), with statistical significance denoted as **** *p* ≤ 0.0001. Statistical analysis was performed using one-way ANOVA followed by Dunnett’s multiple comparison test.

Western blot analysis revealed significant reductions in pro-IL-1β protein levels (31 kDa) across all siRNA-treated groups (Figure 2A). The most pronounced knockdown was achieved by siIL-1β.2, reducing pro-IL-1β protein levels by approximately 85% compared to the siCon-treated control, followed by siIL-1β.1 (75%) and siIL-1β.3 (65%) (Figure 2B).

Enzyme-linked immunosorbent assay (ELISA) measurements confirmed these results, with siIL-1β.2 reducing IL-1β protein concentrations by 83% compared to the control, followed by siIL-1β.1 (78%) and siIL-1β.3 (65%) (Figure 2C). Interestingly, a weak level of IL-1β was detectable even without LPS stimulation (control), suggesting that PMA-induced differentiation alone is sufficient to induce low levels of IL-1β expression (Figure 2C; see also Supplementary Figure S2 for additional control conditions).

These findings demonstrate that siIL-1β.2 is the most effective candidate for endogenous IL-1β knockdown and that PMA slightly induces basal IL-1β even in the absence of LPS.

### 2.3. Nebulization Setup and Cell Viability

**Figure 3:**
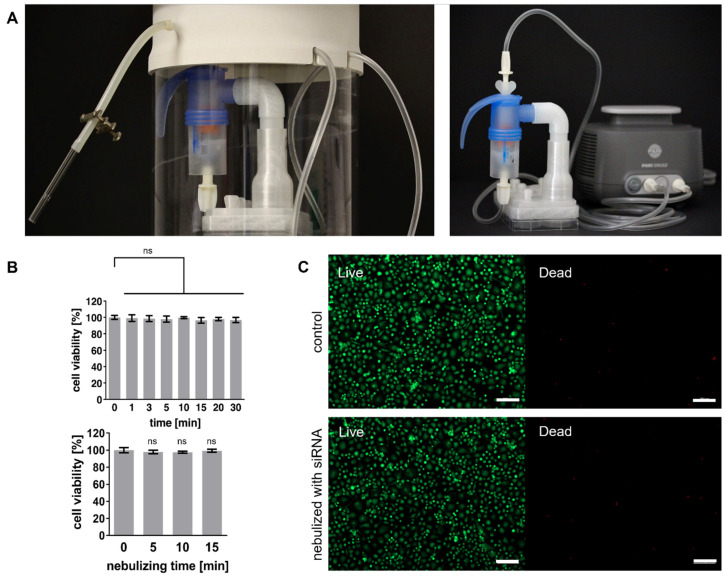
Evaluation of cell viability and nebulization setup for siRNA delivery. (**A**) Custom nebulizer chamber for siRNA delivery, incorporating the PARI SINUS2 nebulizer with 3D-printed adapters for a 6-well plate. The setup includes an aspirator outlet to prevent mist leakage. (**B**) XTT assay results for THP-1 Mφ: (**Top**) Viability after removal of medium for varying time points up to 30 min. (**Bottom**) Viability after nebulization with Opti-MEM for up to 15 min. (**C**) Live-dead staining of THP-1 Mφ: (**Top**) Control cells cultured without nebulization. (**Bottom**) Cells nebulized with 20 nM siRNA (siIL-1β.2) for 5 min. Live cells are indicated in green and dead cells in red. Data are represented as mean ± SD from three independent experiments (n = 3), ns indicates a non-significant difference (*p* > 0.05). Statistical analysis was performed using one-way ANOVA followed by Dunnett’s multiple comparison test. Scale bars = 200 μm.

The siRNA delivery experiments were conducted using a custom-made nebulization chamber to confine aerosols containing siRNA and prevent free-floating particles in the surrounding environment (Figure 3A, left). As a safety measure an additional outlet connected to an aspirator prevented mist leakage. The PARI SINUS2 jet nebulizer, enhanced with 3D-printed adapters (Figure S3), directed aerosolized siRNA solutions onto 6-well plates (Figure 3A, right).

To ensure that the nebulization process and the required removal of culture medium did not adversely affect cell viability due to shear forces, osmotic stress, or dehydration, XTT assays were conducted on THP-1 Mφ. Viability was normalized to untreated controls: submerged cells (0 min medium removal) and non-nebulized cells (0 min). The results confirmed consistently high cell viability of >95% across all time points, even after prolonged incubation without medium for up to 30 min (Figure 3B, top). Further, nebulization with Opti-MEM alone did not compromise cell viability (Figure 3B, bottom), confirming the biocompatibility of the process.

A calibration curve was generated using serial dilutions of blue food dye (Figure S4), yielding a linear relationship between concentration and absorbance at 620 nm (y = 1.65x + 0.02, R^2^ = 0.99). Post-nebulization, the deposited volume in each well was determined via this calibration, with a mean of 30.7 ± 2.4 µL. This corresponded with a deposition efficiency of ~1.5%.

Gel electrophoresis revealed distinct siRNA complexation efficiencies across nitrogen-to-phosphate (N/P) ratios 0–3. Partially complexed siRNA was detectable at N/P ratios ≥ 0.25, while complete complexation occurred at N/P ≥ 1 (Figure S5). Given the potential cytotoxicity of high RNAiMAX concentrations and the practical necessity of minimizing reagent consumption in large-scale applications, the N/P ratio of 0.25 was selected for further nebulization experiments as it represents the lowest detectable siRNA-lipoplex formation while ensuring cost-efficient reagent utilization.

For subsequent nebulization experiments, the best-performing siRNA candidate, siIL-1β.2, was nebulized for 5 min, followed by a 10-min settling period. The required siRNA and transfection reagent concentrations were determined from calibration curves (Figure S4) and complex formation efficiency tests (Figure S5) to achieve a final well concentration of 20 nM siRNA after medium replenishment, ensuring cost-effective usage while minimizing potential cytotoxicity.

To assess potential cytotoxic effects of optimized siRNA nebulization, live-dead staining was performed after nebulization with siIL-1β.2 onto THP-1 Mφ. Staining revealed no significant increase in dead cells (red) compared to the control group, with nearly all cells remaining viable (green) post-nebulization (Figure 3C). These results demonstrate that the nebulization process is non-toxic and preserves cell integrity during siRNA delivery.

### 2.4. siRNA Delivery via Nebulization

**Figure 4:**
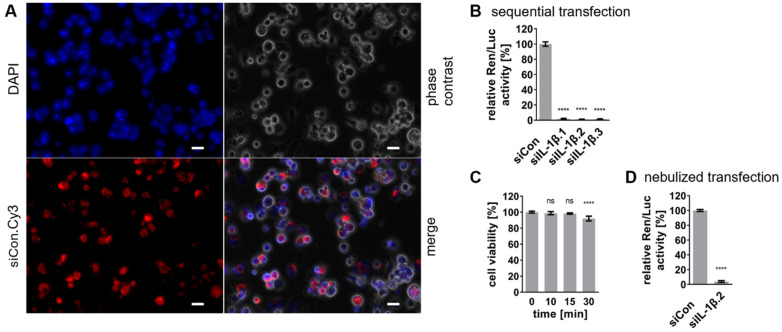
Evaluation of siRNA delivery and functionality post-nebulization. (**A**) Fluorescence microscopy of THP-1 Mφ transfected with fluorescent-labeled siCon.Cy3 via nebulization. Shown are DAPI nuclear staining (blue), phase contrast, siCon.Cy3 signal (red), and the merged overlay. (**B**) Dual-luciferase reporter assay after sequential transfection, in which THP-1 Mφ were first transfected with the reporter plasmid and subsequently with siRNA 6 h later. Relative luciferase activity was normalized to siCon set as 100%. (**C**) Cell viability assessment by XTT assay in HeLa cells following temporary medium removal for up to 30 min. (**D**) Dual-luciferase reporter assay following siRNA delivery via nebulization, normalized to siCon set as 100%. Data are presented as mean ± SD from three independent experiments (n = 3), with statistical significance denoted as **** *p* ≤ 0.0001 and ns, non-significant (*p* > 0.05). Statistical analysis was performed using one-way ANOVA with Dunnett’s multiple comparisons or unpaired Student’s *t*-test. Scale bars = 20 μm.

With the nebulization setup established, siRNA delivery into THP-1 Mφ was preliminarily evaluated using fluorescent-labeled siCon.Cy3. As shown in Figure 4A, DAPI staining confirmed the presence of cells within the field of view, while phase contrast imaging validated the macrophage-like morphology of the differentiated THP-1 cells. Red fluorescence from siCon.Cy3 indicated successful interaction of the siRNA with the cells. The co-localization of siCon.Cy3 red fluorescence within the cells suggests siRNA uptake; however, it does not conclusively demonstrate cytoplasmic delivery or endosomal release, which are critical for effective gene silencing. Transfection efficiency following nebulization was quantified using a Fiji-based macro for single-cell analysis, which determines the proportion of fluorescently labeled cells relative to the total cell population [44]. Analysis of three independent biological replicates revealed a transfection efficiency of 90.0 ± 2.5% (mean ± SD, n = 3), confirming highly efficient cellular uptake of the nebulized siRNA.

### 2.5. siRNA Integrity and Functionality Post-Nebulization

The next step was to evaluate whether the nebulization process affected siRNA integrity and functionality. To this end, DLAs were carried out with sequential transfections. This approach consisted of first introducing reporter plasmid DNA into the cells, followed by siRNA transfection 6 h later. As shown in Figure 4B, sequential transfection resulted in significant knockdown of relative Ren/Luc activity by at least 98% for all siRNAs compared to the negative control (siCon), confirming their functionality.

To determine whether the nebulization process and the required removal of culture medium affected the cell viability of HeLa cells, XTT assays were performed. The XTT assays confirmed that HeLa cell viability remained unaffected for up to 15 min of medium removal, supporting the feasibility of nebulization conditions (Figure 4C).

For nebulization experiments, HeLa cells were seeded and initially transfected with the reporter plasmid. After a 6-h incubation, siIL-1β.2 was delivered via nebulization at a final concentration of 20 nM. Following a 48-h incubation, DLA results demonstrated that siIL-1β.2 retained its silencing efficiency post-nebulization, reducing relative Ren/Luc activity by 96% compared to the siCon control (Figure 4D). This confirms that the nebulization process preserves siRNA functionality.

### 2.6. Knockdown of Endogenously Expressed IL-1β by Nebulized siRNA

Finally, the silencing efficacy of nebulized siIL-1β.2 against endogenously expressed IL-1β was evaluated. For this, THP-1 Mφ were transfected with siIL-1β.2 via nebulization and then stimulated with 100 ng/mL LPS to induce IL-1β production. PMA-differentiated but non-LPS-stimulated cells were included as baseline controls.

**Figure 5:**
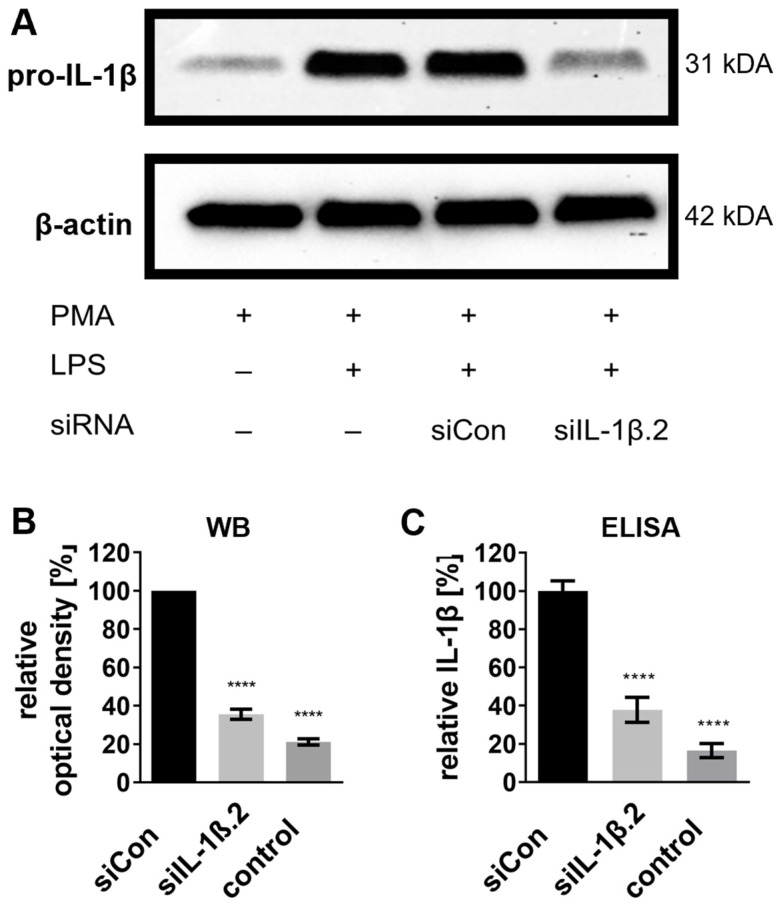
Validation of nebulized siRNA for endogenous IL-1β knockdown in differentiated THP-1 cells. Cells were treated with 100 ng/mL PMA for 72 h to induce differentiation into THP-1 Mφ. Following differentiation, cells were cultured in PMA-free medium for 24 h before transfection with 20 nM siRNA using the PARI SINUS2 Nebulizer System. At 43 h post-transfection, cells were stimulated with 100 ng/mL LPS for 5 h to induce an immune response. Non-stimulated, non-transfected THP-1 Mφ served as the control condition. (**A**) Representative Western blot showing pro-IL-1β protein expression under various treatment conditions, including PMA-induced differentiation, LPS stimulation, and siRNA nebulization. The presence or absence of the respective treatment is indicated as + and −. β-actin served as a loading control to confirm equal protein loading (20 μg per lane). (**B**) Densitometric quantification of pro-IL-1β protein levels from Western blots, normalized to β-actin and expressed relative to siCon (set to 100%). (**C**) Relative IL-1β protein levels quantified by ELISA, normalized to siCon set as 100%. Data are presented as mean ± SD from three independent experiments (n = 3), with statistical significance denoted as **** *p* ≤ 0.0001. Statistical analysis was performed using one-way ANOVA followed by Dunnett’s multiple comparison test.

Western blot analysis showed a significant reduction in pro-IL-1β protein levels following nebulized siIL-1β.2 treatment, achieving approximately 65% knockdown compared to the siCon control (Figure 5A,B). Consistently, ELISA confirmed total IL-1β protein concentrations were reduced by 62% compared to siCon levels upon siIL-1β.2 nebulization (Figure 5C). PMA-treated control cells exhibited baseline IL-1β levels equivalent to 16–21% of the siCon control, strongly suggesting that PMA alone can induce IL-1β expression (see also Supplementary Figure S2).

Taken together, we demonstrated that the designed siRNAs targeting IL-1β exhibit high silencing efficacy with minimal cytotoxicity in THP-1 Mφ. Among the tested candidates, siIL-1β.2 proved to be the most effective, achieving substantial reductions in IL-1β protein levels. Furthermore, we successfully developed a nebulization setup that enables efficient and non-toxic aerosolized delivery of siRNA while preserving both cell viability and siRNA integrity and functionality. Importantly, our findings confirm that nebulized siIL-1β.2 retains its knockdown efficacy. This approach offers an in vitro system to optimize siRNA delivery by nebulization and may support the development of new treatment strategies for pulmonary or localized inflammatory diseases.

## 3. Discussion

This proof-of-concept study demonstrates that siRNA-lipoplexes retain their gene-silencing activity following jet nebulization in a defined in vitro system. The primary objective of this work was to assess whether functional siRNA could be delivered via aerosolization using a standard jet nebulizer and subsequently induce measurable gene knockdown in recipient cells. This objective was achieved, as evidenced by substantial IL-1β protein reduction consistently across complementary Western blot and ELISAs in LPS-stimulated THP-1 Mφ. While nebulized siRNA delivery demonstrated robust gene silencing using a commercially available jet nebulizer, its efficiency can be further enhanced by additional optimization of siRNA-lipoplex formation. The accessibility and ease of use of the nebulizer underscore its potential as a delivery platform for future therapeutic development.

Nebulization provides a non-invasive, targeted approach for siRNA delivery, particularly for respiratory diseases where localized treatment can reduce systemic exposure and off-target effects [31,32,33]. However, the implementation of nebulization in clinically relevant settings remains challenging at early stages of drug development. While several sophisticated in vivo models for pulmonary delivery in mice have been established [45], their translational relevance is limited due to substantial anatomical and physiological differences between murine and human lungs [46]. Human cell-based in vitro systems such as the one presented here offer a complementary approach for early-stage characterization of delivery parameters, consistent with broader efforts to reduce reliance on animal models [36].

Significant advances over recent decades have improved siRNA stability and delivery efficiency to the lungs [47,48,49]. Devices such as nebulizers and inhalers generate aerosols for drug delivery directly to the respiratory system [32,50,51,52]. Clinical trials have evaluated inhaled siRNA (ALN-RSV01) targeting the RSV N gene, demonstrating potential in preventing bronchiolitis obliterans syndrome among lung transplant recipients [53,54,55]. Recently, we developed highly stable and potent siRNAs with broad activity against variants of SARS-CoV-2, targeting conserved regions such as the leader sequence [56,57]. These siRNAs could serve as a basis for future nebulization experiments, expanding our methodological framework of aerosolized RNAi to additional therapeutic targets.

Particle size is critical for efficient pulmonary deposition, with aerosol diameters of 1–5 μm optimally reaching the bronchi and alveoli [31]. The PARI SINUS2 (PARI GmbH, Starnberg, Germany) jet nebulizer used in this study produces aerosols with a mass median aerodynamic diameter of 3 μm, aligning with this optimal range. In addition, jet nebulizers are affordable, easily accessible and already in use by patients with acute and chronic inflammatory conditions [33,58], making them a suitable platform for evaluating nebulization-based siRNA delivery. However, limitations such as dead volume retention and suboptimal aerosolization [50,59,60], can reduce deposition efficiency. Future studies may consider vibrating mesh nebulizers, which offer greater deposition efficiency and reduced medication wastage [33,61,62,63].

Nebulized siRNA delivery successfully achieved substantial and significant IL-1β knockdown in LPS-treated THP-1 Mφ (Figure 5), which were used to model acute inflammatory activation. In the present study, siRNA delivery was performed using RNAiMAX, a well-characterized transfection reagent demonstrated to facilitate efficient endosomal release [64]. The observed knockdown efficiency appears to be influenced by the extent of siRNA-lipoplex formation, as indicated by the N/P ratio of 0.25 selected for nebulization, where only ~40% of siRNA was complexed (Figure S5). Our forward transfection approach employed an N/P ratio of 1, enabling more efficient complexation and thereby enhancing cellular uptake and silencing (Figure 2). Although higher N/P ratios might improve complexation efficiency, they were considered impractical due to cost constraints and the risk of cytotoxicity. Lipoplexes, although widely considered the gold standard for in vitro transfection [26,27], are associated with cytotoxicity, inflammatory potential, and instability in biological fluids [27,28,29,30,65,66], which would limit their utility beyond such settings. Future studies exploring this platform with more biocompatible nanocarriers, such as lipid nanoparticles, ionizable lipids, or poly(lactic-co-glycolic) acid-based hybrid particles approved for drug delivery and biomedical applications [47,52,67,68,69,70,71,72], would represent a natural methodological progression in enhancing siRNA stability, intracellular delivery, and endosomal escape, all critical factors for effective gene silencing [47,52,67,68].

Our approach intentionally employs macrophage-like THP-1 cells as a simple and well-characterized immune-responsive cell system, prioritizing experimental control and reproducibility. This model neither fully recapitulates the complex of the lung microenvironment nor captures cell-type specificity across diverse airway populations. Factors such as mucus barriers, mechanical forces, and interactions with epithelial cells significantly influence siRNA uptake and immune response [31,48,73]. Future studies in primary macrophages, mixed airway cell cultures, and non-immune cell types will be important for assessing cell-type specificity and extending the evaluation of nebulization-based siRNA delivery across diverse pulmonary cell populations. Additionally, three-dimensional (3D) lung models and organ-on-chip systems should be employed to better mimic human lung architecture and biological interactions [74,75,76,77], providing more realistic pulmonary relevance before progressing to in vivo studies. Downstream signaling analyses (e.g., NF-κB pathway modulation, inflammasome activation) will be pursued in follow-up studies to connect delivery efficiency with pathway-level biological responses.

The present work establishes a practical, accessible, and reproducible in vitro platform confirming that siRNA functionality is preserved following jet nebulization. This methodological foundation can be systematically extended to more complex cellular systems, refined with alternative carrier formulations, and ultimately applied to disease-relevant models for respiratory inflammation.

## 4. Materials and Methods

### 4.1. siRNA Design and Synthesis

The siRNA design was done in silico using multiple online computational tools, including Genscript (Nanjing, China), GeneLink RNAi Explorer (Hawthorne, NY, USA), and OligoWalk (University of Rochester Medical Center, Rochester, NY, USA), to consider critical parameters including target sequence accessibility, minimal off-target effects, GC content, thermodynamic stability, and predicted mRNA secondary structure. The online software mFold (Rensselaer Polytechnic Institute, Troy, NY, USA) was used to align the siRNAs with the predicted secondary structure of IL-1β mRNA (Figure S1) to evaluate target accessibility. A total of three siRNA candidates siIL-1β.1, siIL-1β.2, and siIL-1β.3 were selected for further analysis and the absence of seed sequences in the human transcriptome was ensured by the Nucleotide BLAST 2.14.1 (NCBI, Bethesda, MD, USA) program using default parameters. The control siRNA siCon does not match any sequence present in the human genome [42]. The siIL-1β.1–3 targeting the *IL1B* gene were synthesized by Microsynth AG (Balgach, Switzerland). Both siCon and fluorescently labeled siCon.Cy3 were synthesized by Eurofins Genomics (Ebersberg, Germany). All siRNAs and their corresponding sequences are shown in Table 1.

### 4.2. Analysis of siRNA Integrity

To confirm that the selected and ordered siRNAs targeting IL-1β were double-stranded, a urea polyacrylamide gel electrophoresis (PAGE) was performed. The synthesized siRNAs siIL-1β.1–3 were loaded onto the gel alongside control siRNA siCoV6 (Figure S6), which served as a positive control for size, as it is a known functional double-stranded siRNA from previous experiments [56,57]. Since urea-PAGE denatures double-stranded siRNA into single strands, intact siRNA is expected to appear as a single band if both strands are the same length. All samples were mixed with 2× RNA Loading Dye (B0363S, New England Biolabs, Ipswich, MA, USA). A 1 μg aliquot of each siRNA was loaded onto the polyacrylamide–urea gel (3043, Carl Roth GmbH & Co. KG, Karlsruhe, Germany). After running the gel in 1× ROTIPHORESE^®^ TBE buffer (3061, Carl Roth GmbH & Co. KG) for approximately 2 h at 100 V, it was stained with ROTI^®^GelStain (3865, Carl Roth GmbH & Co. KG) for 30 min. The bands were visualized using Molecular Imager Gel Doc XR+ (Bio-Rad Laboratories, Hercules, CA, USA).

### 4.3. Plasmid Construction and Reporter Assay

The silencing activity of the siRNAs was assessed in initial dual-luciferase reporter assays using a vector expressing the firefly and *Renilla* luciferase reporter gene. The target sequences of the respective siRNAs (siIL-1β.1, siIL-1β.2, siIL-1β.3) were synthesized by Thermo Fischer Scientific (Invitrogen, Carlsbad, CA, USA) and sequences were inserted downstream of the *Renilla* luciferase gene of the psiCHECK™ 2 vector (C8021, Promega, Madison, WI, USA) plasmid using the XhoI/NotI restriction sites in the MCS, resulting in psiCHECK™-2 all TS siIL-1β (Figure 1B). The correct insertion and sequence integrity were confirmed by DNA sequencing through LGC Genomics (Berlin, Germany).

### 4.4. Cell Culture

Human cervical HeLa cancer cells (ACC 57, DSMZ, Braunschweig, Germany) were cultured in Dulbecco’s modified Eagle’s medium high glucose (DMEM, L0101, Biowest, Nuaillé, France), 10% fetal bovine serum (FBS, c.c.pro, Oberdorla, Germany), 2 mM L-Glutamine (L-Glu, X0550, Biowest), 1% 100× MEM non-essential amino acids (NEAA, X0557, Biowest), and 1% penicillin/streptomycin (P/S, L0022, Biowest). HeLa cells were seeded at 8 × 10^4^ cells per well in a 24-well plate or 2.5 × 10^5^ cells per well in a 6-well plate.

Human monocytic leukemia cells (THP-1, ACC 16, DSMZ) were cultured in DMEM (Biowest) supplemented with 5% FBS (c.c.pro), 2mM L-Glu (Biowest), 1% NEAA (Biowest), and 1% P/S (Biowest). THP-1 cells were seeded at 1 × 10^5^ cells per well in a 24-well plate, 3 × 10^5^ cells per well in a 12-well plate or 5 × 10^5^ cells per well in a 6-well plate. To induce differentiation of monocytic THP-1 cells into adherent macrophage-like cells (THP-1 Mφ), the cells were treated with 100 ng/mL PMA (phorbol 12-myristate 13-acetate, 524400, Sigma-Aldrich, Merck KGaA, Darmstadt, Germany) for 72 h. Following differentiation, cells were maintained in PMA-free medium for 24 h for further maturation before siRNA transfection. At 43 h post-transfection, cells were stimulated with 100 ng/mL LPS from *Escherichia coli* O111:B4 (L4391, Sigma-Aldrich, Merck KGaA) for 5 h to induce an immune response and IL-1β production. Subsequently, the samples were harvested for the analysis of IL-1β protein expression levels using Western blot and ELISA.

All cells were incubated at 37 °C and 5% CO_2_ in a humidified atmosphere.

### 4.5. Forward Transfection of THP-1 Mφ with siRNA

THP-1 Mφ cells were transfected 24 h after PMA-induced differentiation. The siRNAs were transfected at a concentration of 20 nM using Lipofectamine™ RNAiMAX Transfection Reagent (13778150, Thermo Fisher Scientific, Invitrogen), according to the manufacturer’s protocol. Briefly, cells were seeded and differentiated in culture plates to be 70–80% confluent at the time of transfection. For transfection, Lipofectamine™ RNAiMAX was diluted in Opti-MEM™ I Reduced Serum Medium (11058, Thermo Fisher Scientific, Waltham, MA, USA) and incubated for 5 min at room temperature. Separately, siRNA was diluted in Opti-MEM™. The transfection complexes were prepared using an N/P ratio of 1 by combining equal volumes of diluted RNAiMAX and siRNA solutions (1 μL RNAiMAX per 10 pmol siRNA), followed by 15 min incubation at room temperature to allow complex formation. The siRNA-lipoplexes were then added to the cells, which were incubated at 37 °C for 43 h prior to LPS stimulation.

### 4.6. Transfection of HeLa Cells with Plasmid and siRNA

For forward transfection, HeLa cells were co-transfected 24 h after seeding in 24-well plates with 250 ng of the reporter plasmid psiCHECK™-2 all TS siIL-1β and 20 nM or 40 nM of the respective siRNA using Lipofectamine™ 2000 (11668019, Thermo Fisher Scientific, Invitrogen) according to the manufacturer’s protocol.

For sequential transfection, HeLa cells were first transfected 24 h after seeding in 24-well plates with 250 ng reporter plasmid psiCHECK™-2 all TS siIL-1β using Lipofectamine™ 2000, according to the manufacturer’s instructions. After 5 h, the cell culture medium was changed, and the cells were allowed to rest in fresh medium for another one hour. The cells were then transfected with 20 nM siRNA using Lipofectamine™ RNAiMAX, according to the manufacturer’s instructions.

In both cases, cells were incubated at 37 °C for 48 h after transfection.

### 4.7. Quantification of siRNA Deposition via Nebulization

To quantify siRNA deposition, a surrogate dye approach was employed using Icing Colours—Royal Blue Food Dye (Wilton, Naperville, IL, USA). The dye was chosen for its biocompatibility and distinct absorbance at 620 nm. A calibration curve was established by measuring the absorbance of serial dye dilutions (Figure S4).

For nebulization, a 6-well plate was placed in a custom-designed chamber. A defined volume of dye solution was nebulized using the PARI SINUS2 system (PARI GmbH, Starnberg, Germany) equipped with 3D-printed adapters (Figure S3). After a 5-min aerosolization period, mist was allowed to settle for 10 min to ensure complete deposition. Wells were then rinsed with a defined volume V_rinse_, and the absorbance A_sample_ of the recovered solution was used to calculate the dilution factor D_sample_ derived from the calibration curve. The deposited volume V_deposited_ was then determined as:

V_deposited_ = D_sample_ × V_rinse_

The deposition efficiency (DE) was calculated as:

DE = V_deposited_/V_nebulizer_

where the required nebulizer reservoir fill volume is V_nebulizer_ = 2000 µL.

To achieve the target siRNA concentration (C_target_ = 20 nM = 0.02 µM) in a well volume of V_well_ = 2000 µL after medium replenishment, the required siRNA concentration in the nebulizer reservoir (C_reservoir_) was calculated based on the measured DE:

C_reservoir_ [µM] = (C_target_ × V_well_)/DE × V_nebulizer_

This equation was used to determine the siRNA input concentration in the nebulizer reservoir for each experimental replicate, based on the corresponding measured DE value.

### 4.8. Design and Fabrication of 3D-Printed Nebulization Adapters

The custom 3D-printed adapters were designed using CAD software PTC Creo 7.0 (Parametric Technology Corporation, Boston, MA, USA). The 3D models were then processed using the Bambu Studio 1.9.0 software (Bambu Lab, Shenzhen, China) to generate the G-code for printing. The adapters were fabricated from polylactic acid filament (DasFilament, Emskirchen, Germany) using a Bambu Lab X1C printer (Bambu Lab).

### 4.9. Evaluation of Lipoplex Formation Efficiency

Lipoplex formation was evaluated to determine the minimal volume of Lipofectamine™ RNAiMAX required for efficient siRNA complexation, based on varying N/P ratios (Figure S5). Briefly, lipoplexes were prepared by incubating different volumes of RNAiMAX (µL) in Opti-MEM for 5 min at room temperature followed by the addition of 50 pmol siRNA (siCon or siIL-1β.2) and further incubation for 30 min. The final mixture was supplemented with 15% glycerol (3783, Carl Roth GmbH & Co. KG) prior to loading onto a 2% agarose (3810, Carl Roth GmbH & Co. KG) gel stained with ROTI^®^GelStain (1:20,000 dilution). Electrophoresis was conducted at 120 V for 10 min. The bands were visualized using Molecular Imager Gel Doc XR+.

### 4.10. Transfection of Eukaryotic Cells with siRNA via Nebulization

HeLa cells were seeded in 6-well plates 24 h prior to sequential transfection. First, the cells were transfected with 625 ng of the reporter plasmid psiCHECK™-2 all TS siIL-1β using Lipofectamine™ 2000, according to the manufacturer’s instructions. After a 5 h incubation, cells were replenished with fresh medium and rested for 1 h before nebulization. For siRNA delivery, both HeLa cells and THP-1 Mφ were transfected using the PARI SINUS2 nebulizer in a custom-built chamber equipped with 3D-printed adapters designed specifically for 6-well plates (Figure S3). The chamber incorporated an aspirator outlet to maintain containment and minimize aerosol leakage. Based on deposition efficiency calculations, the nebulizer reservoir was filled with 2 mL of transfection solution containing siRNA complexed with RNAiMAX at N/P 0.25 (previously determined by gel electrophoresis). For transfection, culture medium was first removed from the wells, followed by a 5-min active nebulization period. The aerosols were allowed to settle for 10 min to ensure complete deposition before fresh medium was added to support cell recovery. This standardized protocol was applied consistently to both HeLa cells and THP-1-derived macrophages, with the latter being differentiated using PMA treatment as previously described in the cell culture Section 4.4.

Following nebulization, transfection efficiency was assessed using a Fiji-based single-cell analysis macro [44], which quantifies the fraction of fluorescently labeled cells within the total cell population.

### 4.11. Cytotoxicity Assays

Cell toxicity was determined by measuring LDH release in the cell culture supernatant using the CyQUANT™ LDH Cytotoxicity Assay (C20300, Thermo Fisher Scientific, Invitrogen) according to the manufacturer’s protocol. Furthermore, cell viability was determined using the XTT assay (J61726.MD, Thermo Fisher Scientific), which measures cellular metabolic activity through mitochondrial dehydrogenase-mediated reduction of tetrazolium salt. The XTT working solution was prepared by combining 1 mg/mL XTT in phenol red-free RPMI 1640 (L0505, Biowest) with phenazine methosulfate (PMS; P9625, Sigma-Aldrich, Merck KgaA) at 3.83 mg/mL in a 500:1 volume ratio. This mixture was added to the cells and incubated for 4 h (37 °C, 5% CO_2_). Following incubation, absorbance was measured at 450 nm with a 620 nm reference using a microplate reader (TriStar Multimode Microplate Reader LB942, Berthold Technologies, Bad Wildbad, Germany). To account for background absorbance, control samples were treated with 70% ethanol for 10 min prior to XTT addition.

For cell viability assay by live/dead staining, cells were stained with 1 µM calcein AM (80011, Biozol, Hamburg, Germany) and 1 µM ethidium homodimer-1 (E1169, Thermo Fisher Scientific, Invitrogen) diluted in phenol red-free RPMI for 15 min (37 °C, 5% CO_2_). The samples were imaged by a fluorescence microscope (Zeiss Observer Z1 microscope; Zeiss, Jena, Germany).

### 4.12. Dual-Luciferase Reporter Assay

HeLa cells were either co-transfected or sequentially transfected with siRNA and the reporter plasmid psiCHECK™-2 all TS siIL-1β as described above. The efficacy of siRNAs in silencing was evaluated at 48 h post-transfection by measuring relative luciferase activity using the Dual-Luciferase Reporter Assay System (E1980, Promega), according to the manufacturer’s instructions.

### 4.13. Western Blot

Differentiated and LPS-stimulated THP-1 cells in 6-well plates were washed twice with cold Dulbecco’s phosphate-buffered saline (PBS, L0615, Biowest) 48 h after transfection and lysed in 150 μL RIPA buffer (89900, Thermo Scientific, Pierce™, Rockford, IL, USA) supplemented with Protease and Phosphatase Inhibitor Cocktail (1:100, PPC1010, Sigma-Aldrich, Merck KGaA). Protein concentration was determined using the Pierce™ bicinchoninic acid (BCA) protein quantitation kit (23227, Thermo Fisher Scientific) according to the manufacturer’s instructions. Protein separation was performed by sodium dodecyl sulfate (SDS)-PAGE using Mini-PROTEAN TGX Stain-Free gels (4568093, Bio-Rad Laboratories). Protein samples were prepared for running by adding 1× NuPAGE™ LDS Sample Buffer (NP0007, Thermo Fisher Scientific, Invitrogen) and heating at 70 °C for 10 min. Subsequently, 20 μg of the samples and 5 μL of CozyHi^TM^ Prestained Protein Ladder (PRL0202, highQu GmbH, Kraichtal, Germany) were loaded onto the gel. Proteins were transferred from the SDS gel to a PVDF membrane (3010040001, Roche Diagnostics, Mannheim, Germany) using the PerfectBlue™ Semi-Dry Electroblotter (Peqlab Biotechnologie GmbH, Erlangen, Germany) at 70 mA for 70 min. Blocking was performed for 1 h at room temperature in 5% milk powder (T145, Carl Roth GmbH & Co. KG) blocking buffer. The membrane was incubated overnight at 4 °C with a primary antibody against IL-1β (12242, 1:1000, Cell Signaling Technologies, Danvers, MA, USA) diluted in blocking buffer. After three washes with PBS containing Tween, the membrane was incubated with an HRP-conjugated secondary antibody goat anti-mouse (31430, 1:10,000, Thermo Scientific, Pierce™) for 1 h at room temperature. Chemiluminescence detection was performed using the Pierce ECL Western Blotting Substrate Kit (32209, Thermo Scientific, Pierce™) according to the manufacturer’s instructions. Images were captured using the ChemiDoc™ MP Imaging System (Bio-Rad Laboratories) and analyzed with Image Lab Software 6.1 (Bio-Rad Laboratories). For β-actin detection as a loading control, the membrane was stripped using Western Blot Stripping Buffer (ab282569, Abcam, Cambridge, UK), washed, re-blocked, and incubated with β-actin antibody conjugated to HRP (HCA147P, 1:1,000, Bio-Rad Laboratories). Relative optical density analysis was performed using ImageJ 1.54h software (National Institutes of Health, Bethesda, MD, USA) to quantify IL-1β signal intensities, which were normalized to β-actin.

### 4.14. ELISA

Differentiated and LPS-stimulated THP-1 cells were treated with 5 mM adenosine triphosphate (ATP; A6419, Sigma-Aldrich, Merck KGaA) for 30 min. Supernatants were collected to quantify the release of IL-1β using the Human IL-1β uncoated ELISA kit (88-7261-88, Thermo Fisher Scientific, Invitrogen) according to the manufacturer’s protocol. Briefly, wells in a 96-well plate were coated with capture antibody and incubated overnight at 4 °C. Following washing and blocking steps, samples and standards were added to the wells and incubated for 2 h at room temperature. After further washing, detection antibodies and Avidin-HRP were sequentially added with incubation steps. The reaction was developed using TMB substrate, stopped, and absorbance was measured at 450 nm with a reference at 620 nm using the TriStar Multimode Microplate Reader. All samples were run in triplicate, and the standard curve was used to quantify IL-1β concentrations.

### 4.15. Statistical Analysis

All data are presented as the mean ± standard deviation (SD) from at least three independent experiments (n = 3). The statistical significance was analyzed by one-way analysis of variance (ANOVA) or unpaired Student’s *t*-test using GraphPad Prism 8 Software (Boston, MA, USA) where applicable. *p*-values > 0.05 were considered non-significant (ns). Statistical significance is indicated as * *p* ≤ 0.05, ** *p* ≤ 0.01, *** *p* ≤ 0.001, or **** *p* ≤ 0.0001.

## 5. Conclusions

In conclusion, this study provides a methodological proof-of-concept demonstrating that commercially available nebulizers can effectively deliver siRNA while maintaining biological activity in macrophage-like THP-1 cells. Using an accessible, off-the-shelf nebulizer, we validated siRNA stability and IL-1β knockdown following aerosolization. Our presented in vitro model serves as a practical platform for systematic characterization and optimization of siRNA formulations and aerosolization parameters. The acute inflammatory model employed here cannot directly assess therapeutic potential for chronic inflammatory diseases or malignancies. Validation in primary macrophages, mixed airway cell cultures, appropriate disease models, and physiologically relevant systems will be necessary to progress toward preclinical development of inhaled RNAi therapies.

## Figures and Tables

**Table 1:** siRNA sequences used in this study.

siRNA	Nucleotide Sequence [5′–3′]	Application
siCon s siCon as	UUC UCC GAA CGU GUC ACG U[dTdT] ACG UGA CAC GUU CGG AGA A[dTdT]	RNA interference negative control
siCon.Cy3 s siCon.Cy3 as	UUC UCC GAA CGU GUC ACG U[dTdT] ACG UGA CAC GUU CGG AGA A[dTdT]	RNA interference fluorescently labelled negative control
siIL-1β.1 s siIL-1β.1 as *	CCA AAG AAG AAG AUG GAA A[dTdT] UUU CCA UCU UCU UCU UUG G[dTdT]	RNA interference against IL-1β
siIL-1β.2 s siIL-1β.2 as *	GCG UGU UGA AAG AUG AUA A[dTdT] UUA UCA UCU UUC AAC ACG C[dTdT]	
siIL-1β.3 s siIL-1β.3as *	CUU UGA AGA AGA ACC UAU C[dTdT] GAU AGG UUC UUC UUC AAA G[dTdT[	

as, antisense. s, sense. * binds to target mRNA.

## Data Availability

The raw data supporting the conclusions of this article will be made available by the authors on request.

## Supplementary Materials

The following supporting information can be downloaded at: https://www.mdpi.com/article/10.3390/ijms27062915/s1.

## Abbreviations

The following abbreviations are used in this manuscript:

ANOVA	analysis of variance
ATP	adenosine triphosphate
DE	deposition efficiency
DLA	dual-luciferase assay
DMEM	Dulbecco’s modified Eagle’s medium
ELISA	enzyme-linked immunosorbent assay
FBS	fetal bovine serum
IL-1β	Interleukin-1 beta
LDH	lactate dehydrogenase
L-Glu	L-glutamine
LPS	lipopolysaccharide
N/P	nitrogen-to-phosphate
NEAA	non-essential amino acid
P/S	penicillin/streptomycin
PAGE	polyacrylamide gel electrophoresis
PBS	phosphate-buffered saline
PMA	phorbol 12-myristate 13-acetate
RNAi	RNA interference
SDS	sodium dodecyl sulfate
siRNA	small interfering RNA
THP-1 Mφ	THP-1-derived macrophage-like cells
XTT	2,3-bis-(2-methoxy-4-nitro-5-sulfophenyl)-2H-tetrazolium-5-carboxanilide
