# Supplementary material for: Functional siRNA Delivery via Jet Nebulization: Proof-of-Concept IL-1ß Silencing in Macrophage-like THP-1 Cells
Source: Int J Mol Sci. 2026 Mar 23;27(6):2915. doi: 10.3390/ijms27062915 (PMC13026121; doi:10.3390/ijms27062915)
Supplement: Supplementary file 1 [file ijms-27-02915-s001.zip › ijms-4121329-supplementary.pdf]

Supporting Information

Nebulized siRNA Delivery for Targeted Knockdown of IL-1β in Macrophage-Like Cells

Duy Bao Tran Nguyen[1], Ahmed S. M. Ali[1], Dongwei Wu[1], Johanna Berg[1], Daniel C. Lauster[2], Jens Kurreck[1,] and Beatrice Tolksdorf[1,*]*

[1] Department of Applied Biochemistry, Institute of Biotechnology, Technische Universität Berlin, 10623 Berlin, Germany.

[2] Institute of Pharmacy, Biopharmaceuticals, Freie Universität Berlin, 12169 Berlin, Germany

Correspondence should be addressed to Beatrice Tolksdorf (tolksdorf@tu-berlin.de) and Jens Kurreck (jens.kurreck@tu-berlin.de).

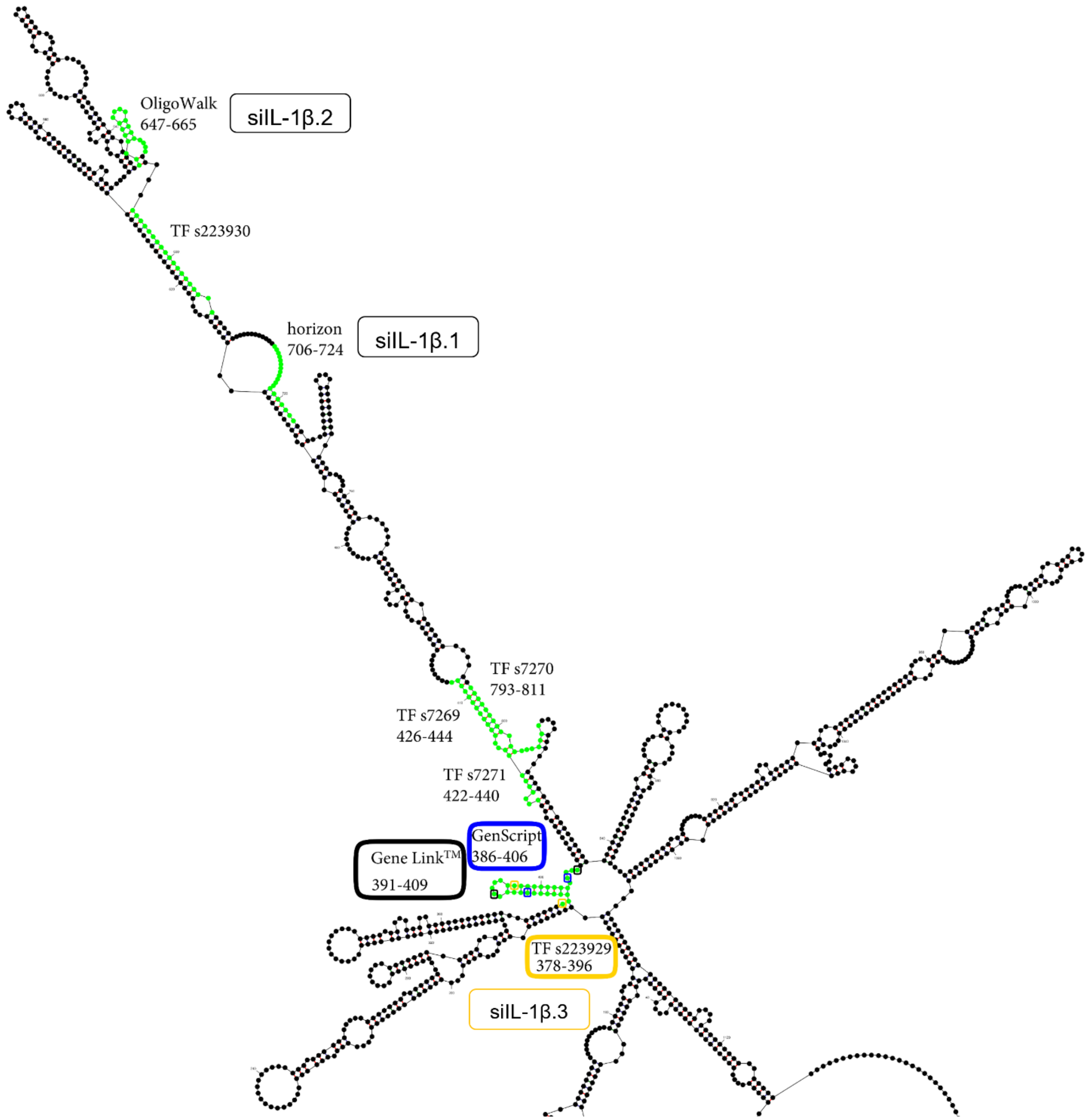

Figure S1. Magnified region of predicted IL-1β mRNA secondary structure with siRNA target sites. A zoomed-in region of the IL-1β mRNA secondary structure, predicted using the mFold tool, is displayed. Highlighted in green are regions that have been identified as potential targets for gene silencing by various siRNA design tools and manufacturers. Several tools, including GenScript and GeneLink, as well as a commercial siRNA from Thermo Fisher Scientific (TF), predicted overlapping target sites within a hairpin structure. This figure focuses on the key regions of interest rather than the entire mRNA sequence.

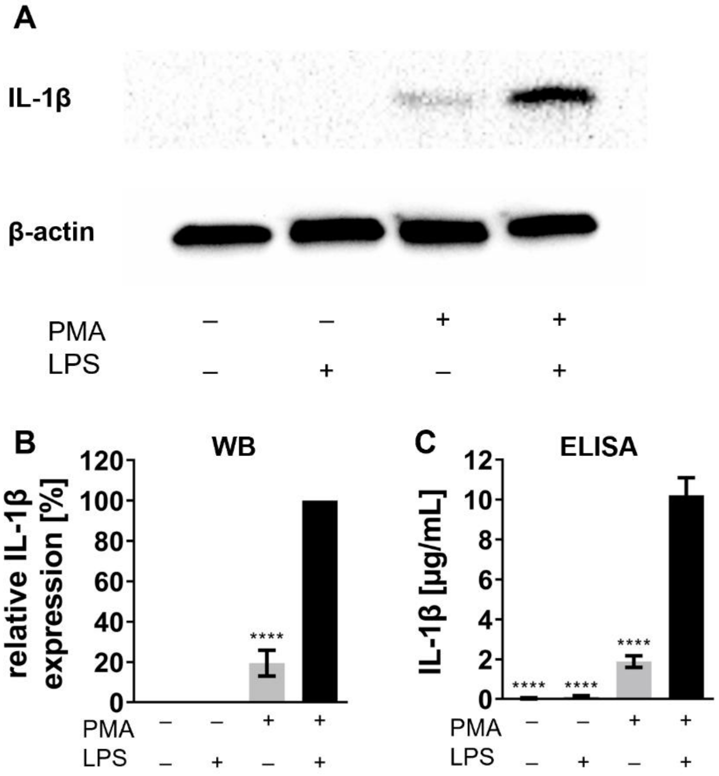

Figure S2. IL-1β protein expression in THP-1 cells across four conditions: untreated (–PMA, –LPS), LPS-stimulation without differentiation (–PMA, +LPS), PMA-induced differentiation without LPS stimulation (+PMA, –LPS), and LPS stimulation of differentiated cells (+PMA, +LPS). **(A)** Western blot with β-actin as loading control. **(B)** Densitometric quantification of IL-1β protein levels from Western blots was normalized to β-actin, with differentiated and stimulated cells (+PMA, +LPS) set as 100%. **(C)** Absolute IL-1β protein levels quantified by ELISA (µg/mL). Data are presented as mean ± SD from three independent experiments (n = 3).

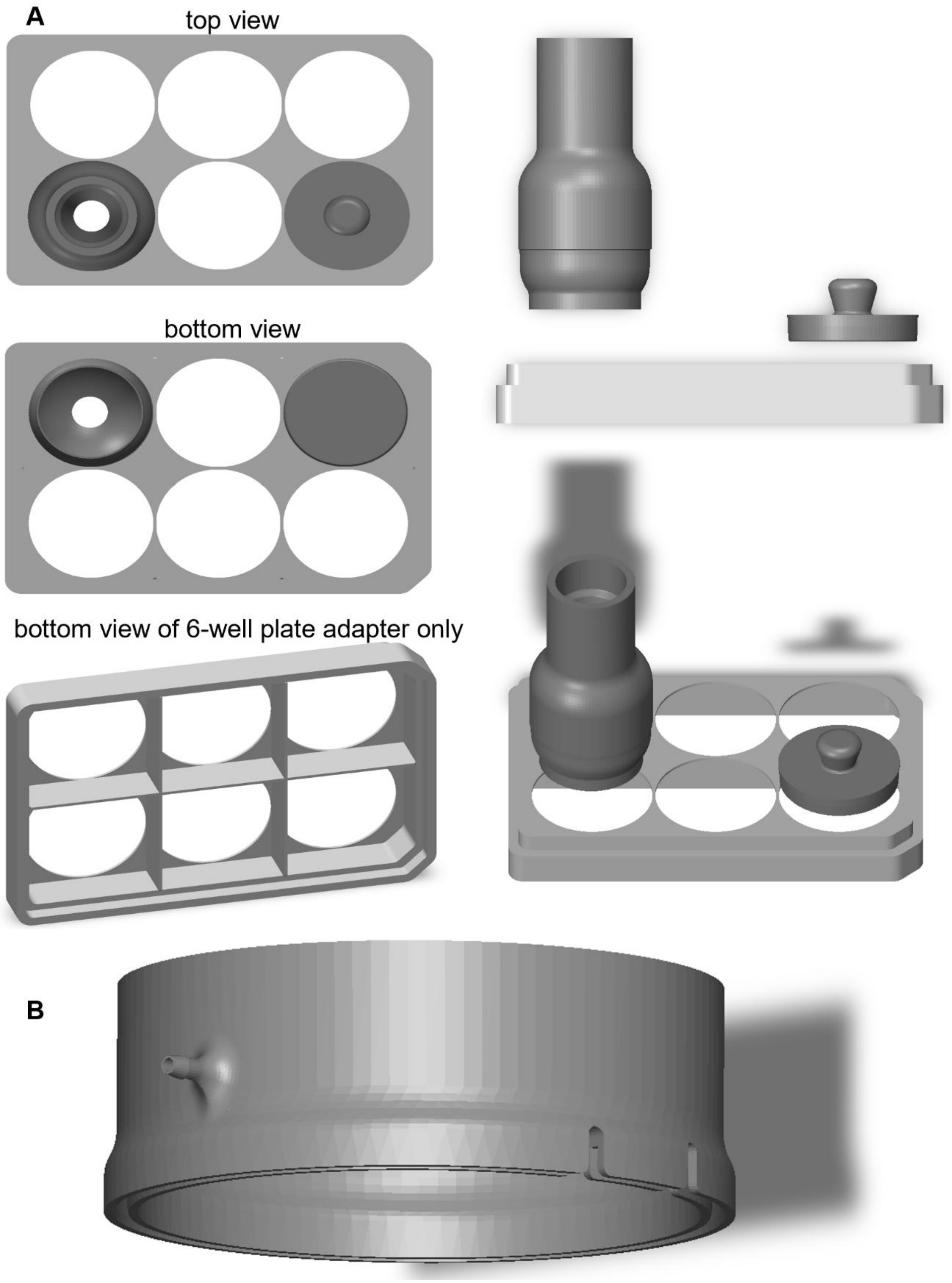

Figure S3. Illustration of 3D-printed adapters for nebulization. (**A**) Different perspectives of a custom-made nebulizer setup designed to optimize siRNA delivery in a 6-well plate format. The PARI SINUS2 Nebulizer System was modified with printed adapters to route the aerosol flow directly onto the wells. (**B**) Chamber extension with an additional outlet for an aspirator to prevent mist leakage and cutouts for nebulizer tubing.

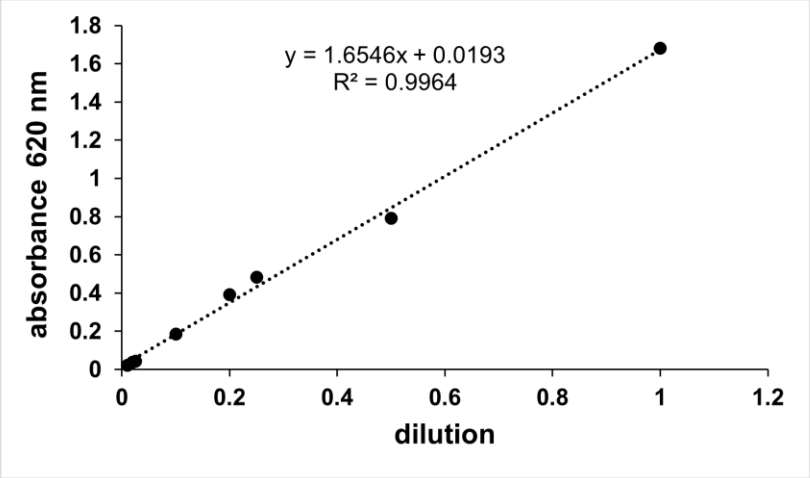

Figure S4. Calibration curve used for quantification of dye deposition in wells. The curve was generated by measuring the absorbance of serial dilutions of the blue food dye at 620 nm. The linear regression equation is y = 1.6546x + 0.0193 with R^2 = 0.9964.

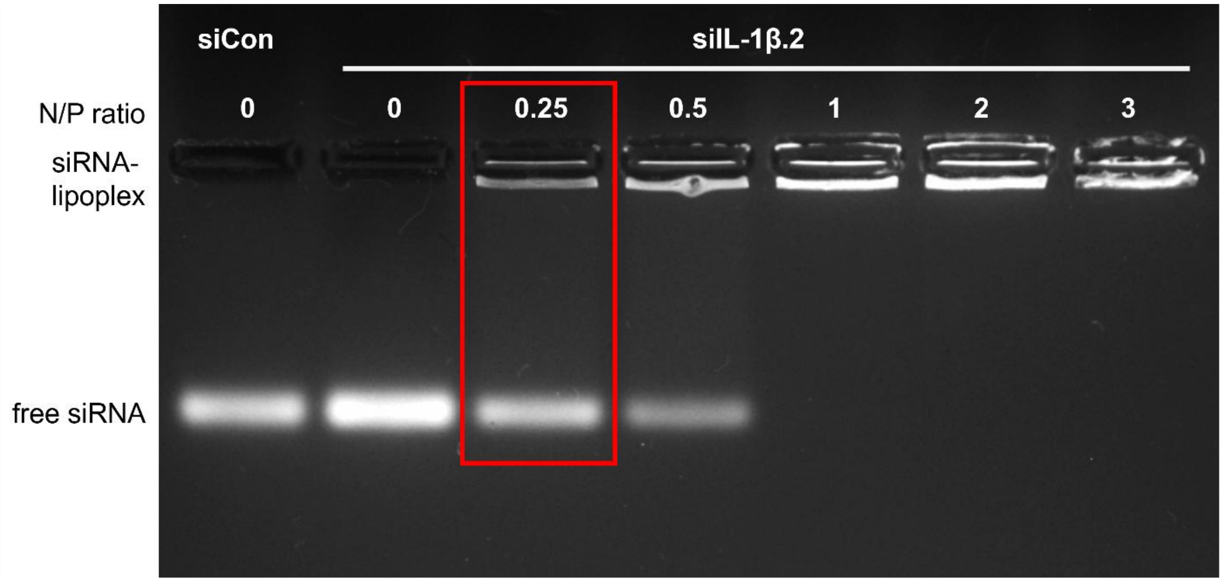

Figure S5. siRNA-lipoplex formation efficiency**.** Agarose gel electrophoresis of siRNA/RNAiMAX complexes prepared at different nitrogen-to-phosphate (N/P) ratios, where ratios represent the volume of RNAiMAX (μL) per 10 pmol siRNA. Each lane contained 50 pmol siRNA. Electrophoresis was performed in a 2% agarose gel at 120 V for 10 min. The red box denotes the selected N/P 0.25 ratio for nebulization experiments, representing the minimal effective complexation that balanced efficiency with cost considerations.

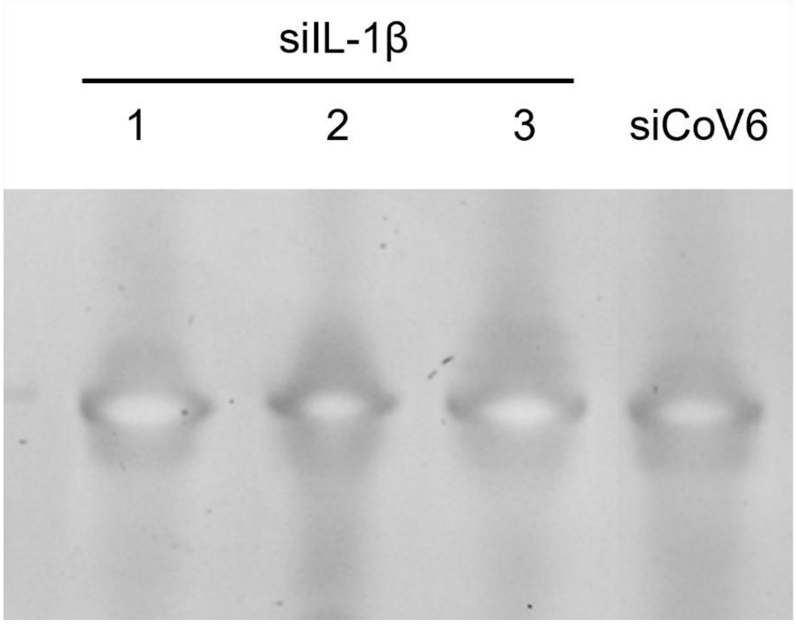

Figure S6. Verification of siRNA double-stranded integrity using urea gel electrophoresis. Urea polyacrylamide gel electrophoresis of siRNA samples stained with ROTI®GelStain. The synthesized siIL-1β candidates (siIL-1β.1, siIL-1β.2, siIL-1β.3) were loaded onto the gel alongside control siRNA siCoV6. SiCoV6 serves as positive size control for double stranded siRNAs. Under denaturing conditions, double stranded siRNA separates into single strands of equal length and migrates as a single band if the siRNA is intact.
